# *Syphacia obvelata* antigens alter the FOXP3/RORɣt expression balance in isolated peripheral blood mononuclear cells of IBD patients

**DOI:** 10.1038/s41598-025-06619-0

**Published:** 2025-07-24

**Authors:** Abbas Amin, Niloofar Taghipour, Mohammad Rostami-Nejad, Foroogh Alborzi Avanaki, Reyhaneh Jafarshad, Seyyed Javad Seyyed Tabaei, Nariman Mosaffa

**Affiliations:** 1 Department of Parasitology and Mycology, School of Medicine, Shahid Beheshti University of Medical Sciences, Tehran, Iran; 2 Celiac Disease and Gluten Related Disorders Research Center, Research Institute for Gastroenterology and Liver Diseases, Shahid Beheshti University of Medical Sciences, Tehran, Iran; 3 Imam Khomeini Hospital GI Ward, Tehran University of Medical Sciences, Tehran, Iran; 4 Present Address: Imam Ali Hospital Complex, Alborz University of Medical Sciences, Karaj, Iran; 5 Department of Immunology, School of Medicine, Shahid Beheshti University of Medical Sciences, Tehran, Iran

**Keywords:** *Syphacia obvelata*, IBD, FOXP3, RORɣt, Inflammatory bowel disease, Parasitology

## Abstract

Inflammatory Bowel Disease (IBD) arises from disrupted interactions among intestinal microbiota, epithelial cells, and the immune system, which are influenced by genetic and environmental factors. A critical factor in IBD pathogenesis is the balance between FOXP3^+^ regulatory T cells (Tregs) and RORγt^+^ Th17 cells; a decreased Treg/Th17 ratio can lead to inflammation. *Syphacia obvelata* may help modulate immune responses by promoting Th2 responses and enhancing Treg populations, potentially impacting FOXP3 and RORγt expression and aiding IBD management. In this study, peripheral Blood Mononuclear Cells **(**PBMCs) from 6 IBD patients were treated with *S. obvelata* antigens (ES-Ag, S-Ag, and ES/S-Ag) for 24 h. Optimal concentrations and time points were determined via the MTT assay. Total RNA was extracted, cDNA was synthesized, and RT-qPCR was performed using FOXP3 and RORγt primers. The gene expression fold changes and FOXP3/RORγt ratios were compared to the control group. Results showed that FOXP3 expression was increased significantly after treatment with all *S. obvelata* antigens, including ES-Ag, S-Ag, and ES/S-Ag, whereas the expression of RORγt decreased significantly in just two groups, including ES-Ag and ES/S-Ag. Eventually, the FOXP3/RORγt gene expression fold change ratio was increased significantly after 24 h of exposure to *S. obvelata* antigens. Our study indicates that the ES-Ag, S-Ag, and ES/S-Ag of *S. obvelata*, along with their combination, can increase the FOXP3/RORɣt ratio in PBMCs from patients with IBD, suggesting anti-inflammatory effects. This finding offers hope that these antigens could be an eye-opener for developing future strategies for IBD treatment.

## Introduction

Inflammatory Bowel Disease (IBD) encompasses a spectrum of inflammatory conditions affecting the large and small intestines, with Crohn’s disease and ulcerative colitis as the principal types^1^. The pathogenesis of IBD is characterized by disrupted interactions among intestinal microbiota, epithelial cells, and the mucosal immune system, leading to chronic inflammation. Both genetic and environmental factors play crucial roles in influencing these interactions and determining the susceptibility to IBD^2^. The analysis of three studies on IBD epidemiology reveals critical trends. The age-standardized incidence rate (ASIR) of IBD increased from 4.22 per 100,000 in 1990 to 4.45 in 2021, with regional variations. East Asia and China saw significant increases, while regions with higher socio-demographic indices, like high-income North America and Central Europe, experienced decreased incidence and mortality. Despite rising incidence, the age-standardized mortality rate (ASMR) and disability-adjusted life years (DALYs) have declined, indicating a reduction in disease burden. In 2019, there were 405,000 new IBD cases worldwide, with mortality and DALYs decreasing. Predictions indicate new cases and deaths will continue to fall from 2020 to 2050. Annual incidence rates vary geographically, with Europe and Oceania higher than Asia, the Middle East, and South America. The burden of IBD is increasing among children, adolescents, and older adults due to environmental factors like tobacco use, antibiotic exposure, and diet. Protective factors include breastfeeding and a diet rich in fruits, vegetables, and fiber, while smoking is linked to worse outcomes in Crohn’s disease^3–5^.

The balance between FOXP3^+^ regulatory T-cells (Tregs) and RORγt^+^ Th17 cells is critical in maintaining immune homeostasis, particularly in the context of IBD^6^. A decreased FOXP3^+^ Tregs to Th17 ratio has been associated with developing IBD^7^suggesting that an imbalance may contribute to the pathogenesis of the disease. The induction of Tregs is crucial for controlling excessive inflammation, and a higher FOXP3 expression appears to be associated with better clinical outcomes in IBD. Conversely, elevated levels of Th17 have been implicated in driving pro-inflammatory responses that exacerbate the disease^7^. RORɣt is primarily expressed in Th17 cells and promotes the differentiation of thymocytes to pro-inflammatory Th17 cells^8^. The Th17/Treg ratio is directly associated with the expression of RORγt, STAT3, and FOXP3 to differentiate corresponding cells^9^.

Helminths, particularly nematodes, can enhance mucosal immunity and mitigate inflammation through various mechanisms, including promoting mucus secretion and modulating immune responses. Research indicates that nematode infections can stimulate Th2 responses and regulatory T cell (Treg) populations, leading to increased production of anti-inflammatory cytokines such as IL-10 ^10–12^. It has also shown that secretions of some parasitic worms induce the de novo expression pathway of FOXP3 and TGF-β^13^. Additionally, other studies acknowledge the suppression of Th17-based responses in infection by parasitic worms^14^. *Syphacia obvelata*, a nematode commonly found in laboratory animals, has shown promise due to its ability to induce a Th2 immune response and modulate immune tolerance in experimental models^15–17^. This study aims to investigate the effects of *S. obvelata* antigens on the balance of FOXP3/RORγt expression in isolated peripheral blood mononuclear cells in IBD patients. We examined how this ratio is altered in vitro, assessing the gene expression of key markers associated with regulatory and pro-inflammatory T-cell populations. Further understanding the mechanisms underlying these changes may provide insights into novel therapeutic strategies for managing IBD.

## Materials and methods

All experimental protocols were approved by Shahid Beheshti University’s Institutional Review Board (IRB) (IR.SBMU.MSP.REC.1401.62), and all methods were carried out in accordance with relevant guidelines and regulations. Additionally, this study is reported in accordance with the ARRIVE guidelines (Animal Research: Reporting of In Vivo Experiments).

### Preparation of *S. obvelata* adult worms

Laboratory mice infected with *S. obvelata* (Shahid Beheshti University of Medical Sciences animal house) were identified using the Scotch tape method, and the infection was confirmed by examining the morphology of parasite eggs. Infected male mice, aged 5 to 7 weeks, were housed with uninfected mice (Royan Institute animal house) for two weeks to facilitate the transmission of the infection. Following this period, infected mice were euthanized by cervical dislocation, and their ceca were excised.

Cecum contents were suspended in PBS solution, and *S. obvelata* specimens were isolated under a stereo-microscope based on their morphological characteristics. The worms were washed 5 to 10 times with PBS solution, depending on contamination levels. The molecular method was employed to confirm the morphological identification of the worms. DNA was extracted from selected worms using the DNA EXTRACTION Kit (DNP™) (Sinnaclon, Tehran, Iran) following the manufacturer’s instructions. PCR amplification was conducted using *S. obvelata*-specific primers^18^.

### Preparation of the *S. obvelata* antigens

Excretory-secretory (ES) antigens of worms were prepared using the Bungiro et al. study protocol with some modifications. Briefly, adult worms were incubated in sterile PBS containing penicillin (100 IU/ml) and streptomycin (100 µg/ml) at 37 °C for 8 to 12 h at a concentration of approximately 100 worms per milliliter. Post-incubation, the worms were removed, and the PBS solution containing the ES antigens was centrifuged at 3300 g for 15 min at 4 °C. The supernatant was filtered through a 0.22 μm Millipore syringe filter, and the protein concentration was measured using the BCA protein assay (Bio Basic Inc.). The filtered ES-Ag was subsequently stored at -80 °C^19^.

For somatic antigens (S-Ag), adult worms obtained from the previous procedure were suspended in 0.05 M TRIS-HCl buffer (pH 7.5) at an approximate concentration of 100 worms per milliliter. The suspension was homogenized in a glass tissue homogenizer (Witeg, Germany) on ice for 10 min. Following homogenization, the mixture was centrifuged at 8000 g for 2 min at 4 °C, and the supernatant containing the S-Ag was collected^20^. This supernatant was filtered as previously described, and the protein concentration was determined using the BCA protein assay (Bio Basic Inc., Canada). The remaining supernatant was stored at -80 °C.

### Isolation of hPBMCs and MTT assay

To optimally determine the antigen potency to stimulate and influence the peripheral blood mononuclear population, we took samples from healthy volunteers. We performed in vitro stimulation to ensure that it was safe. Blood samples from healthy individuals were collected into heparin-sodium-coated tubes and transported to the Dept. of Parasitology and Mycology, Shahid Beheshti University of Medical Sciences. Human peripheral blood mononuclear cells (hPBMCs) were isolated using density gradient separation with Histoprep 1.077 (BAG Diagnostics, Germany), and cell viability was evaluated using Trypan blue (0.4%) staining. To determine optimal antigen concentration and exposure duration for PBMC stimulation, cells were treated with *S. obvelata* antigens at 5, 10, and 20 µg/ml concentrations for 24, 48, 72, and 96 h. An MTT assay was performed to identify optimal conditions. hPBMCs were seeded in 96-well plates in complete RPMI 1640 medium (Biosera, France), with antigens added and incubated at 37 °C in a 5% CO2 and humidified atmosphere. After incubation, the MTT reagent was added, and cells were incubated for four more hours. Formazan crystals were solubilized with acidic isopropanol, quantified using a spectrophotometer at 550 nm, and the stimulation index (SI) was calculated.

### IBD patients

In general, the criteria for including patients in this study were as follows: a confirmed diagnosis of IBD (active phase regardless of their disease activity index) based on endoscopic evaluation and gastroenterologist validation, an age range of 15 to 40 years, and a negative screening for hepatitis B and C, HIV, active and chronic tuberculosis, cytomegalovirus, as well as a negative stool test for *Clostridium difficile*. Conversely, the exclusion criteria comprised health conditions and histories that could confound the results. These included: a prior history of intestinal resection related to IBD, perianal diseases, a history of severe myocardial infarction, neurodegenerative disorders, pregnancy during the time of blood sampling, any history of colon cancer, intestinal strictures, abscesses, fistulas, sepsis, any confirmed active or recent parasitic infection, and the use of anti-TNF medications^21–25^. Additionally, all patients with recent protozoan/helminthic disease that might interfere with the study results were excluded from the study.

### hPBMCs stimulation

According to the MTT assay previously described, the optimal antigen concentrations were determined to be 20 µg/ml for ES-Ag and 5 µg/ml for S-Ag and ES/S-Ag, with the ideal antigen exposure time being 24 h. Peripheral blood mononuclear cells (PBMCs) were isolated from 15 to 20 ml of peripheral blood collected from 6 patients diagnosed with IBD (five ulcerative colitis and one Crohn’s disease). The IBD condition of these patients was identified and confirmed by the gastroenterologist using the inclusion and exclusion criteria outlined in the previous section. All patients were in the active phase of the disease, regardless of the disease activity index (DAI). This rigorous selection process ensured a well-defined patient population for our study. The isolated PBMCs were seeded at a density of 1 × 10^6^ cells/ml in RPMI 1640 medium (Gibco) supplemented with 10% Fetal Bovine Serum (FBS), penicillin (100 U/ml), and streptomycin (100 µg/ml) in 12-well cell culture plates. The specific concentrations of *S. obvelata* antigens were then added to designated wells. The plates were incubated at 37 °C with 5% CO2 and 95% humidity for 24 h.

### Quantitative PCR

Total RNA from human Peripheral Blood Mononuclear Cells (hPBMCs) was extracted using the manual TRIZOL (NTA, Iran) method, following the GENE ALL RNA extraction protocol. The isolated total RNA from each sample was used as a template for complementary DNA (cDNA) synthesis, employing a first-strand cDNA synthesis kit (Yekta Tajhiz, Iran) for SYBR Green Assay. Quantitative PCR was conducted on the StepOnePlus system (ABI) targeting RORɣt, FOXP3, and the housekeeping gene GAPDH. Each reaction was performed in a 10 µl volume, consisting of 400 ng cDNA, 5 µl SYBRGreen PCR master mix (Ampliqon, Denmark), nuclease-free water, and 1 pmol of both forward and reverse primers (each 10 pmol*/*µL). The sequences of both gene primers are shown in the Table 1.

**Table 1:** Primer sequences for the target genes.

Target gene	Accession No.	Primers
RORɣt	NM_005060.4	F: 5’-ACTCAAAGCAGGAGCAATGGAA-3’ R: 5’-AGTGGGAGAAGTCAAAGATGGA-3’
FOXP3	NM_014009.4	F: 5’-CACCTGGAAGAACGCCATCC-3’ R: 5’-CTCATCCACGGTCCACACAG-3’
GAPDH	NM_002046.7	F: 5’-CCACTCCTCCACCTTTGACG-3’ R: 5’-CCACCACCCTGTTGCTGTAG-3’

The amplification protocol was initiated by DNA polymerase activation at 95 °C for 10 min. This was followed by 45 cycles of denaturation at 95 °C for 10 s, annealing at 60 °C for 25 s, and extension at 72 °C for 25 s. A melting cycle was then performed, consisting of 10 s at 95 °C, 1 min at 60 °C, and 10 s at 95 °C. All PCR reactions were executed in duplicate and validated by the presence of a single peak in the melt curve analysis.

### Statistical analysis

The One-way ANOVA test was used to compare the ratios of mRNA expression before and after the exposure of PBMCs to the *S. obvelata* antigens. *P* ≤ 0.05 was considered statistically significant. All statistical analyses were performed using Prism version 9.5 for Microsoft Windows software (GraphPad, San Diego, USA).

### Ethical statement

Our research adhered to ethical standards for integrity and participant welfare, following the Declaration of Helsinki, and was approved by Shahid Beheshti University’s Institutional Review Board (IRB) under code IR.SBMU.MSP.REC.1401.62. After a detailed explanation of the study’s objectives, procedures, risks, and benefits, all participants provided informed consent.

We prioritize participant confidentiality; data were anonymized, securely stored, and accessed only by authorized personnel.

We disclosed potential conflicts of interest; funding sources did not influence study design, data collection, analysis, interpretation, or reporting.

In publishing our findings, we ensure transparency and accuracy, acknowledging limitations and the need for further research. Our commitment to ethical research advances knowledge and enhances patient care.

## Results

**Fig. 1:**
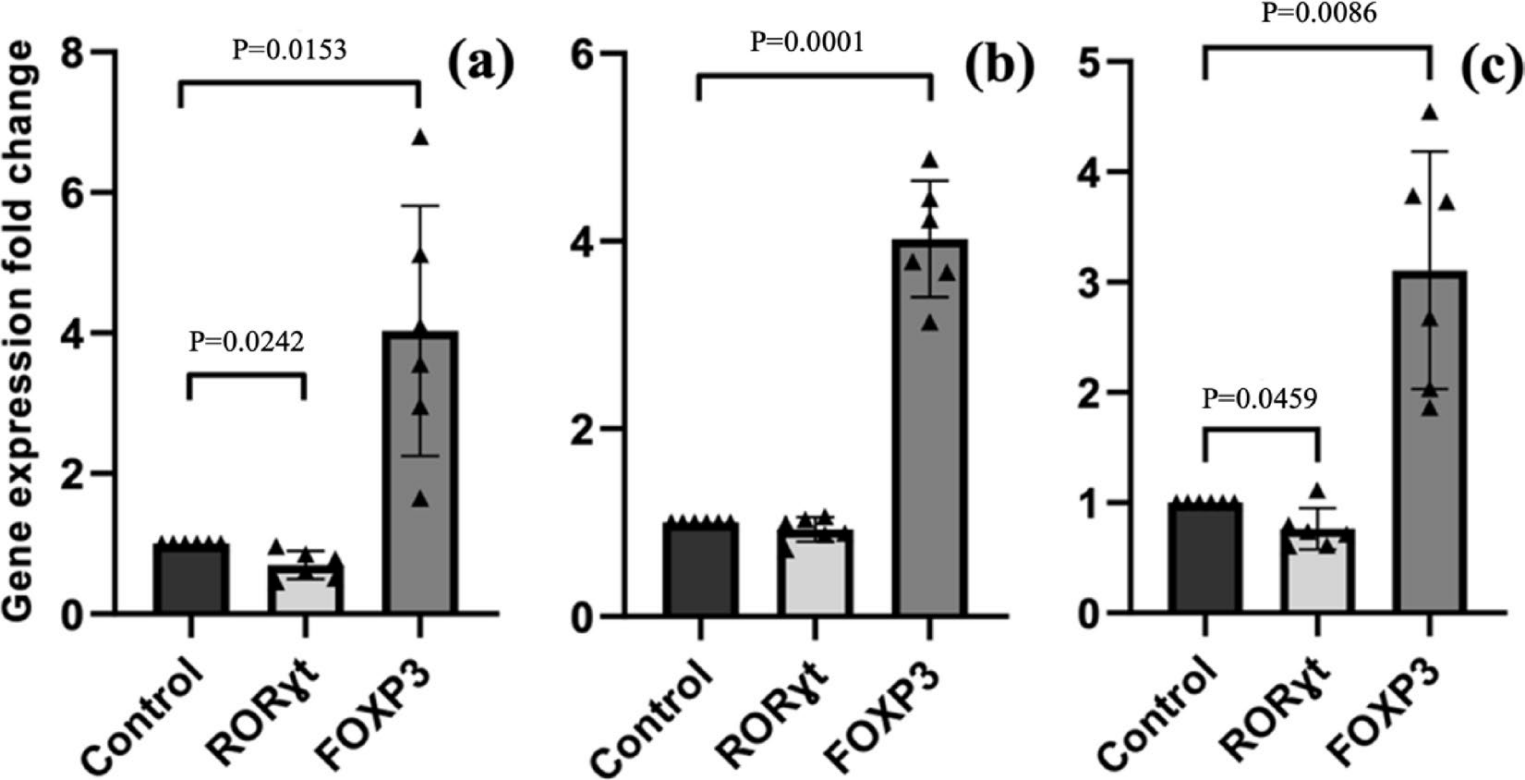
Comparison of FOXP3 and RORɣt gene expression in PBMCs isolated from IBD patients exposed to *S. obvelata* antigens. (**a**) ES-Ag exposure, (**b**) S-Ag exposure, and (**c**) ES/S-Ag exposure.

We examined the expression of two master regulator genes, FOXP3 and RORγt, in peripheral blood mononuclear cells using real-time PCR after exposure to *S. obvelata* antigens. Briefly, based on the MTT assay results, the optimal exposure duration for all antigens (ES-Ag, S-Ag, and ES/S-Ag) was determined to be 24 h. Furthermore, the most effective antigen concentrations were established as 20 µg/mL for ES-Ag and 5 µg/mL for S-Ag and ES/S-Ag. We evaluated the RT-qPCR results by calculating the fold changes in gene expression for FOXP3 and RORɣt relative to the housekeeping gene GAPDH in peripheral blood mononuclear cells (PBMCs) at the 24-hour time point. This analysis was conducted using the relative expression software tool (REST©), and data were expressed as mean ± S.E.M. For each individual with IBD, the experimental PBMC groups (exposed to *S. obvelata* ES-Ag, S-Ag, and ES/S-Ag) were compared to a control group of PBMCs without exposure to any antigen. The gene expression fold change levels of these two master regulator genes after treatment with *S. obvelata* antigens are presented in the Table 2. As shown, FOXP3 expression was increased significantly after treatment with all *S. obvelata* antigens, including ES-Ag, S-Ag, and ES/S-Ag (Fig. 1a-c), whereas the expression of RORγt decreased significantly just in two groups, including ES-Ag and ES/S-Ag (Fig. 1a-c ).

**Table 2:** Gene expression fold change values ​​for FOXP3 and ROR genes after exposure of PBMCs from IBD patients to *S. obvelata* antigens expressed as mean ± se.

Exposure	FOXP3 GEFC^a^		RORɣt GEFC	
	Mean ± SE	*p*-value	Mean ± SE	*p*-value
ES-Ag	4.03 ± 0.727	0.0153	0.695 ± 0.082	0.0242
S-Ag	4.024 ± 0.253	0.0001	0.927 ± 0.053	0.3534
ES/S-Ag	3.107 ± 0.440	0.0086	0.764 ± 0.076	0.0459

(^a^): gene expression fold change)

**Fig. 2:**
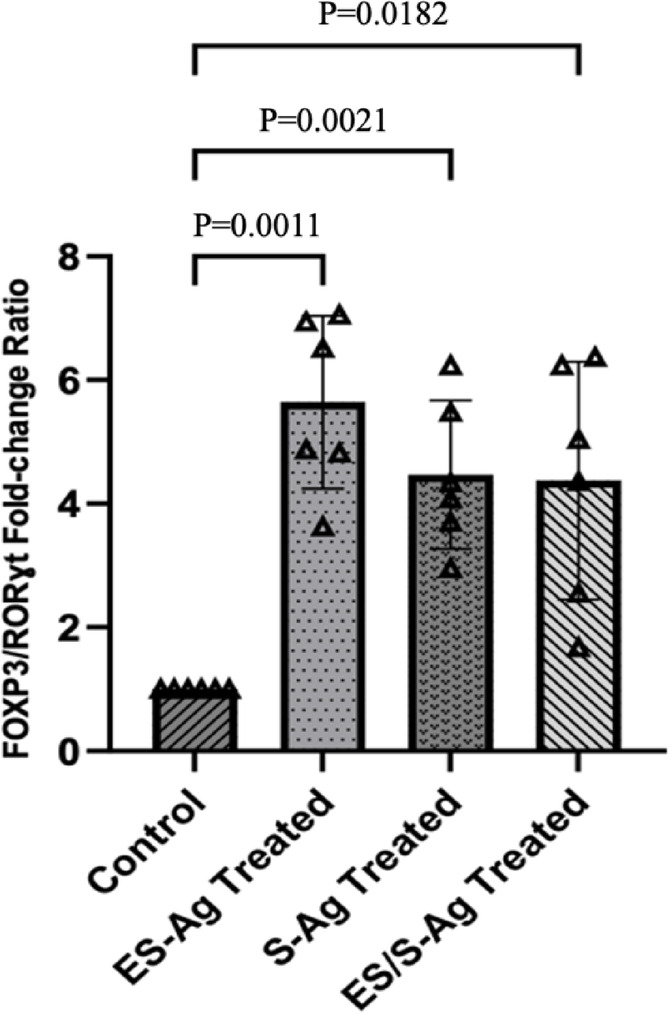
FOXP3/RORɣt gene expression fold changes in PBMCs after treatment with antigens of *S. obvelata* after 24 h.

The gene expression fold change ratios of the two genes were calculated, and the relative changes after ES-Ag, S-Ag, and ES/S-Ag treatment were determined and listed in Table 3. As shown, the FOXP3/RORγt gene expression fold change ratio significantly increased after 24 h of exposure to all three *S. obvelata* antigens (Fig. 2).

**Table 3:** FOXP3/RORɣt values after exposure of PBMCs from IBD patients to *S. obvelata* antigens expressed as mean ± SE.

Exposure	FOXP3/RORɣt ratio	
	(Mean ± SE)	*p*-value
ES-Ag	5.641 ± 0.570	0.0011
S-Ag	4.469 ± 0.49	0.0021
ES/S-Ag	4.373 ± 0.784	0.0182

## Discussion

Parasitic worms employ various strategies to survive the host’s immune system, primarily by producing enzymes that alter or suppress the host’s immune response. These enzymes vary based on the parasite type and its location in the host, leading to unique immune evasion mechanisms for each parasite. Numerous studies support this, with recent research aiming to use worm-derived components to manage harmful immune responses, especially in autoimmune diseases.

In autoimmune diseases like IBD, the Treg cells to Th17 cells ratio is a crucial indicator of disease status, and a decreased ratio flags inflammatory conditions^6^. Previous inconsistencies in measuring Th17 and Treg cell levels might be resolved by directly evaluating their regulatory markers gene expression, RORɣt, and FOXP3, respectively, in the PBMCs of patients^26–31^.

Immunomodulatory properties of *S. obvelata* infection have been previously demonstrated by potent induction of Th2 responses and suppression of allergic reactions in *Mus musculus*^32^. Taghipour et al. found that infection of IBD mouse models with *S. obvelata* improved disease symptoms, induced Th2 immune responses, and modulated immune tolerance in experimental models^15^. Based on these findings, we investigated the effects of excretory-secretory (ES-Ag) and somatic antigens (S-Ag), as well as their combination (ES/S-Ag), on the expression of the master regulators FOXP3 and RORɣt, and the balance between them, which is a marker of inflammatory status in autoimmune diseases like IBD.

Before discussing the findings of the current study, the potential distinct expression of FOXP3 and RORγt in the gut versus PBMCs in UC and CD prompted us to review existing literature on this topic. Overall, the population of CD4^+^CD25^+^FOXP3^+^ Tregs in the peripheral blood of patients with both forms of IBD is reduced^33,34^likely due to their migration to inflamed intestinal regions^35,36^. In one study, patients with active UC exhibited significantly lower peripheral blood Treg percentages compared to healthy controls, and a further decline in circulating Tregs was observed with increasing disease severity^37^. However, the correlation between this reduction and disease severity has been contested in other studies^33^. Furthermore, the presence of Tregs in the mucosal compartment does not necessarily attenuate inflammation, as their functional capacity appears impaired in IBD^38^.

Conversely, a separate study reported markedly elevated RORγt mRNA levels in PBMCs from patients with active UC compared to healthy individuals and those with Crohn’s disease, alongside an increased frequency of RORγt-expressing Th17 cells in the colonic tissue of UC patients^39^. Nevertheless, conflicting data from both animal and human studies have failed to corroborate this upregulation^40,41^. Collectively, these observations indicate that while reduced FOXP3^+^ Tregs and elevated RORγt^+^ Th17 cells in the peripheral blood of IBD patients are frequently reported, these changes may arise from alternative mechanisms, such as cell migration to inflamed sites or methodological variations across studies. This underscores the possibility that in vitro investigations of lymphocyte differentiation, specifically, the enhancement of Tregs or suppression of Th17 cells in response to external stimuli (e.g., pharmacological agents or parasitic antigens), may provide a robust experimental framework for evaluating the immunomodulatory effects of such interventions.

In the present study, 24 h after exposure of six IBD patients’ PBMCs to the *S. obvelata* antigens, FOXP3 gene expression increased significantly across all antigen groups, including ES-Ag, S-Ag, and ES/S-Ag. This aligns with findings on IBD mouse models infected with adult *S. obvelata*, where the number of FOXP3^+^ Treg cells in mesenteric lymph nodes was significantly higher than in control mice^15^. Furthermore, Taghipour et al. reported that FOXP3 expression in mesenteric lymph nodes (MLN) and Peyer’s patches of IBD mouse models, initially reduced, significantly increased post-infection with *S. obvelata* eggs^17^. These consistent results suggest that natural *S. obvelata* infection in IBD mouse models, as well as exposure to the worms’ extracted products and enzymes, can reproduce anti-inflammatory properties in IBD mouse models and PBMCs isolated from IBD patients in vitro, respectively.

Based on our study results, treatment with ES-Ag achieved statistically significant outcomes in both reducing RORγt expression and elevating FOXP3 expression. In contrast, while S-Ag demonstrated greater efficacy in upregulating FOXP3 expression compared to ES-Ag, it did not achieve a statistically significant reduction in RORγt expression. Mirroring our expectations, ES/S-Ag significantly reduced RORγt expression compared to S-Ag alone, aligning with the hypothesis that the inclusion of ES-Ag enhances Th17 pathway suppression. Furthermore, the p-value for FOXP3 upregulation in the ES/S-Ag group reflects an intermediate effect, positioned between the outcomes observed for the individual antigens (ES-Ag and S-Ag). These observations collectively suggest that while S-Ag may preferentially drive FOXP3 induction (potentially via innate receptor activation), ES-Ag contributes critical immunomodulatory signals to suppress RORγt, synergizing with S-Ag in the mixed-antigen formulation.

ES-Ags are bioactive molecules actively released by parasitic nematodes during infection, including proteases, glycoproteins, and immunomodulators (e.g., TGF-β mimics or cytokine homologs). These antigens often manipulate host immunity to promote parasite survival by inducing regulatory T cells (Tregs), Th2 polarization, or anti-inflammatory cytokines like IL-10 ^42^. In contrast, S-Ags are structural components derived from the parasite’s body (e.g., cuticular proteins, intracellular enzymes) that are typically released upon parasite death. These antigens are more likely to trigger pro-inflammatory responses (Th1/Th17) and antibody-mediated defenses due to their recognition as “non-self” by host pattern recognition receptors (PRRs) like Toll-like receptors (TLRs)^43^. While our results completely mirrored the inherent features of the mentioned characteristics of the ES-Ags, the aberrant results from *S. obvelata* S-Ag were surprising. A plausible mechanism for the immunomodulatory behavior of S-Ag may reside in its structural components, such as glycoproteins and heat shock proteins, which could activate TLR2/4 receptors on antigen-presenting cells (APCs). This interaction may subsequently induce the secretion of anti-inflammatory cytokines, including IL-10 and TGF-β, thereby promoting regulatory immune responses^44–47^. Notably, studies have demonstrated the anti-inflammatory effects of somatic antigens from certain helminths, such as *Fasciola hepatica*, *F. gigantica*, and *Trichinella spiralis* in autoimmune diseases, including IBD. These findings align with our observations regarding S-Ag, thereby corroborating the capacity of helminth-derived antigens to modulate immune responses in autoimmune pathologies^48–50^.

Despite numerous studies examining the Treg/Th17 ratio in various autoimmune diseases as an indicator of inflammatory status, limited research has explored the impact of parasitic antigens on altering the FOXP3/RORɣt expression ratio. In this study, the increased FOXP3 expression alongside reduced RORɣt expression resulted in a significant increase in the FOXP3/RORɣt ratio across all antigen groups compared to control cells, suggesting reduced inflammation.

Tuxun et al. found that cystic echinococcosis patients exhibited increased Treg cell numbers and related cytokines, along with elevated FOXP3 levels, while Th17 cells and their associated factors, including RORɣt, showed moderate reductions. This imbalance in the Th17/Treg ratio is a mechanism by which parasites evade the host immune system^51^. A qualitative comparison of this study’s results with our findings indicated agreement between these studies.

Chao Yan et al. investigated the percentage changes of Th17 and Treg cells and their impact on disease severity in BALB/c mice infected with *Clonorchis sinensis*. Their findings showed that while the Treg/Th17 ratio decreased during the first 14 days compared to controls, it significantly increased on days 28 and 56 ^52^. Compared to ours, the initial changes observed in their study could stem from parasite type and infection site, which likely trigger intense inflammatory responses that evolve into chronic immune responses and fibrosis, eventually reducing Th17 cells and increasing Tregs at later time points.

The present study faced several challenges that imposed notable limitations. Due to the stringent inclusion criteria, particularly the requirement for patients to be medication-naïve, which was essential to minimize confounding effects, only six eligible patients were recruited over five months. Of these, one had Crohn’s disease, while the remaining five were diagnosed with ulcerative colitis (UC). While the study’s design, which compared each patient’s PBMCs exposed to helminth antigens to their own untreated PBMCs (self-controlled model), mitigated the impact of the small sample size by minimizing inter-individual variability and enhancing statistical power^53,54^the limited cohort precluded meaningful statistical comparisons between Crohn’s disease and UC. Given the distinct immunological mechanisms underlying these two conditions^55,56^future studies with larger sample sizes are warranted to explore such differences.

As emphasized earlier, this study focused specifically on “antigen-induced changes in IBD patients,” which adequately supported our primary hypothesis. However, we recommend that future research compare baseline immune parameters (e.g., cytokine ratios) between healthy individuals and IBD patients to clarify the disease-specific effects of these antigens.

Although numerous in vitro studies, including ours, have investigated the effects of antigens on PBMCs isolated from patient and healthy donor blood^57–60^translating in vitro findings to clinical relevance requires validation through in vivo models and clinical trials. Thus, we are aiming to utilize the antigens tested here in future animal models to assess their potential therapeutic benefits.

While measuring FOXP3 and RORɣt expression provides valuable insights, these markers alone cannot comprehensively capture functional Treg activity or the balance between pro-inflammatory (Th17) and regulatory responses. To address this, complementary assays would be prioritized in our future investigations:

- Cytokine profiling (e.g., IL-10, TGF-β for Tregs; IL-17 for Th17);
- Protein and cytokine expression analysis of inflammatory/anti-inflammatory mediators (e.g., IFN-ɣ, IL-10, TGF-β);
- Flow cytometry to quantify CD4^+^CD25^+^FOXP3^+^ Tregs and Th17 cells;
- Suppression assays (co-culturing antigen-treated PBMCs with effector T cells), the gold standard for assessing Treg functionality.

Additionally, reduced RORɣt expression may reflect changes in non-Th17 cells (e.g., ILC3s). Flow cytometry could resolve this ambiguity, though prior studies suggest helminth antigens preferentially suppress Th17 differentiation while promoting Treg expansion^61^. Thus, although contributions from other RORγt^+^ cells cannot be definitively excluded, the observed shifts in the FOXP3/RORγt ratio are likely driven by antigen-mediated modulation of T-cell pathways.

While our work and others confirm the immunoregulatory potential of *S. obvelata*, the precise mechanisms linking its antigens to FOXP3 elevation and RORγt suppression, particularly regarding receptors and signal transduction, require targeted experimental validation. This gap presents a critical avenue for future research.

Conclusively, our study suggests that the excretory-secretory and somatic products of *S. obvelata*, as well as their combination, can elevate the FOXP3/RORɣt ratio in PBMCs of IBD patients, indicating anti-inflammatory changes. While these promising results were observed in human cells, further studies are necessary to confirm the efficacy of these parasitic antigens and enzymes in modulating human immune cells effectively.

## Data Availability

The datasets used and/or analyzed during the current study available from the corresponding author on reasonable request.

## References

[1] Talley, N. *Clinical examination: A systematic guide to physical dagnosis* (Elsevier Australia, 2018).

[2] Loscalzo, J. et al. *H1arrison’s principles of internal medicine, Twenty-first edition ***1** (McGraw Hill LLC, 2022).

[3] Caron, B., Honap, S. & Peyrin-Biroulet, L. Epidemiology of inflammatory bowel disease across the ages in the era of advanced therapies. *J. Crohns Colitis*. **18**, ii3–ii15 (2024). 39475082 10.1093/ecco-jcc/jjae082 PMC11522978

[4] Zhou, J. L. et al. Trends and projections of inflammatory bowel disease at the global, regional and National levels, 1990–2050: a bayesian age-period-cohort modeling study. *BMC Public. Health*. **23**, 1–12 (2023). 38097968 10.1186/s12889-023-17431-8 PMC10722679

[5] Lin, D. et al. Global, regional, and National burden of inflammatory bowel disease, 1990–2021: insights from the global burden of disease 2021. *Int J. Colorectal Dis* **39**, (2024). 10.1007/s00384-024-04711-x PMC11380638 39243331

[6] Yan, J., Luo, M., Chen, Z. & He, B. The function and role of the Th17/Treg cell balance in inflammatory bowel disease. *J Immunol Res* 1–8 (2020). (2020). 10.1155/2020/8813558 PMC7755495 33381606

[7] Eastaff-Leung, N., Mabarrack, N., Barbour, A., Cummins, A. & Barry, S. Foxp3 + regulatory T cells, Th17 effector cells, and cytokine environment in inflammatory bowel disease. *J. Clin. Immunol.* **30**, 80–89 (2010). 19936899 10.1007/s10875-009-9345-1

[8] Ivanov, I. I. et al. The orphan nuclear receptor RORγt directs the differentiation program of Proinflammatory IL-17 + T helper cells. *Cell* **126**, 1121–1133 (2006). 16990136 10.1016/j.cell.2006.07.035

[9] Motavalli, R. et al. Altered Th17/Treg ratio as a possible mechanism in pathogenesis of idiopathic membranous nephropathy. *Cytokine* **141**, 155452 (2021). 33571932 10.1016/j.cyto.2021.155452

[10] Weinstock, J. V. & Elliott, D. E. Helminths and the IBD hygiene hypothesis. *Inflamm. Bowel Dis.* **15**, 128–133 (2009). 18680198 10.1002/ibd.20633

[11] Urban, J. F. et al. The importance of Th2 cytokines in protective immunity to nematodes. *Immunol. Rev.* **127**, 205–220 (1992). 1354652 10.1111/j.1600-065x.1992.tb01415.x

[12] Li, Z. et al. The phenotype and function of naturally existing regulatory dendritic cells in nematode-infected mice. *Int. J. Parasitol.* **41**, 1129–1137 (2011). 21827765 10.1016/j.ijpara.2011.06.008

[13] Grainger, J. R. et al. Helminth secretions induce de Novo T cell Foxp3 expression and regulatory function through the TGF-β pathway. *J. Exp. Med.* **207**, 2331–2341 (2010). 20876311 10.1084/jem.20101074 PMC2964568

[14] Walsh, K. P., Brady, M. T., Finlay, C. M., Boon, L. & Mills, K. H. G. Infection with a helminth parasite attenuates autoimmunity through TGF-β-Mediated suppression of Th17 and Th1 responses. *J. Immunol.* **183**, 1577–1586 (2009). 19587018 10.4049/jimmunol.0803803

[15] Taghipour, N. et al. Syphacia obvelata: A new hope to induction of intestinal immunological tolerance in C57BL/6 mice. *Korean J. Parasitol.* **55**, 439–444 (2017). 28877578 10.3347/kjp.2017.55.4.439 PMC5594727

[16] Taghipour, N. et al. Potential treatment of inflammatory bowel disease: A review of helminths therapy. *Gastroenterol. Hepatol. Bed Bench* **7** 9–16 Preprint at (2014). 10.22037/ghfbb.v7i1.510 PMC4017549 25436093

[17] Taghipour, N. et al. Immunomodulatory effect of syphacia obvelata in treatment of experimental DSS-induced colitis in mouse model. *Sci Rep* **9**, (2019). 10.1038/s41598-019-55552-6 PMC6911064 31836772

[18] Parel, J. D. C., Galula, J. U. & Ooi, H. K. Characterization of rDNA sequences from syphacia obvelata, syphacia muris, and aspiculuris tetraptera and development of a PCR-based method for identification. *Vet. Parasitol.* **153**, 379–383 (2008). 18374491 10.1016/j.vetpar.2008.02.001

[19] Bungiro, R. D. Jr., Solis, C. V., Harrison, L. M. & Cappello, M. Purification and molecular cloning of and immunization with Ancylostoma ceylanicum Excretory-Secretory protein 2, an immunoreactive protein produced by adult hookworms. *Infect. Immun.* **72**, 2203 (2004). 15039344 10.1128/IAI.72.4.2203-2213.2004 PMC375217

[20] M, C., LP, C., PJ, H. & P, M. & Ancylostoma factor Xa inhibitor: partial purification and its identification as a major hookworm-derived anticoagulant in vitro. *J. Infect. Dis.* **167**, 1474–1477 (1993). 8501344 10.1093/infdis/167.6.1474

[21] Sorrentino, D., Nguyen, V., Henderson, C. & Bankole, A. Therapeutic drug monitoring and clinical outcomes in immune mediated diseases: the missing link. *Inflamm. Bowel Dis.* **22**, 2527–2537 (2016). 27575494 10.1097/MIB.0000000000000867

[22] Danese, S., Fiorino, G. & Reinisch, W. Review article: causative factors and the clinical management of patients with crohn’s disease who lose response to anti-TNF-α therapy. *Aliment. Pharmacol. Ther.* **34**, 1–10 (2011). 21539588 10.1111/j.1365-2036.2011.04679.x

[23] Benevento, G. et al. Diagnosis and assessment of crohn’s disease: the present and the future. *Expert Rev. Gastroenterol. Hepatol.* **4**, 757–766 (2010). 21108595 10.1586/egh.10.70

[24] Gomollón, F. et al. 3rd European Evidence-based consensus on the diagnosis and management of crohn’s disease 2016: part 1: diagnosis and medical management. *J. Crohns Colitis*. **11**, 3–25 (2017). 27660341 10.1093/ecco-jcc/jjw168

[25] Magro, F. et al. Third European Evidence-based consensus on diagnosis and management of ulcerative colitis. Part 1: definitions, diagnosis, Extra-intestinal manifestations, pregnancy, Cancer surveillance, surgery, and Ileo-anal pouch disorders. *J. Crohns Colitis*. **11**, 649–670 (2017). 28158501 10.1093/ecco-jcc/jjx008

[26] Tada, Y. et al. The balance between Foxp3 and Ror-γt expression in peripheral blood is altered by Tocilizumab and abatacept in patients with rheumatoid arthritis. *BMC Musculoskelet. Disord* **17**, (2016). 10.1186/s12891-016-1137-1 PMC4947268 27421886

[27] Pesce, B. et al. Effect of interleukin-6 receptor Blockade on the balance between regulatory T cells and T helper type 17 cells in rheumatoid arthritis patients. *Clin. Exp. Immunol.* **171**, 237–242 (2013). 23379428 10.1111/cei.12017 PMC3569529

[28] Guggino, G. et al. Targeting IL-6 signalling in early rheumatoid arthritis is followed by Th1 and Th17 suppression and Th2 expansion. *Clin. Exp. Rheumatol.* **32**, 77–81 (2014). 24429356

[29] Samson, M. et al. Brief report: Inhibition of interleukin-6 function corrects Th17/Treg cell imbalance in patients with rheumatoid arthritis. *Arthritis Rheum.* **64**, 2499–2503 (2012). 22488116 10.1002/art.34477

[30] Kikuchi, J. et al. Peripheral blood CD4(+)CD25(+)CD127(low) regulatory T cells are significantly increased by Tocilizumab treatment in patients with rheumatoid arthritis: increase in regulatory T cells correlates with clinical response. *Arthritis Res. Ther* **17**, (2015). 10.1186/s13075-015-0526-4 PMC4332922 25604867

[31] Álvarez-Quiroga, C. et al. CTLA-4-Ig therapy diminishes the frequency but enhances the function of Treg cells in patients with rheumatoid arthritis. *J. Clin. Immunol.* **31**, 588–595 (2011). 21487894 10.1007/s10875-011-9527-5

[32] Jackson, J. A. et al. Immunomodulatory parasites and toll-like receptor-mediated tumour necrosis factor alpha responsiveness in wild mammals. *BMC Biol.* **7**, 16 (2009). 19386086 10.1186/1741-7007-7-16 PMC2685781

[33] Tiwari, V. et al. CD4 + CD25 + FOXP3 + T cell frequency in the peripheral blood is a biomarker that distinguishes intestinal tuberculosis from crohn’s disease. *PLoS One*. **13**, e0193433 (2018). 29489879 10.1371/journal.pone.0193433 PMC5830992

[34] Wang, Y., Liu, X. P., Zhao, Z., Bin, Chen, J. H. & Yu, C. G. Expression of CD4 + forkhead box P3 (FOXP3) + regulatory T cells in inflammatory bowel disease. *J. Dig. Dis.* **12**, 286–294 (2011). 21791023 10.1111/j.1751-2980.2011.00505.x

[35] Jalalvand, M. et al. Blood regulatory T cells in inflammatory bowel disease, a systematic review, and meta-analysis. *Int. Immunopharmacol.* **117**, 109824 (2023). 36827916 10.1016/j.intimp.2023.109824

[36] Ma, Y. H. et al. Increased CD4 + CD45RA-FoxP3low cells alter the balance between Treg and Th17 cells in colitis mice. *http://www Wjgnet Com/*. **22**, 9356–9367 (2016). 10.3748/wjg.v22.i42.9356 PMC5107699 27895423

[37] Gong, Y. et al. The Th17/Treg Immune Imbalance in Ulcerative Colitis Disease in a Chinese Han Population. *Mediators Inflamm* 7089137 (2016). 10.1155/2016/7089137 PMC4763012 26977120

[38] Ban, H. et al. Increased number of FoxP3 + CD4 + regulatory T cells in inflammatory bowel disease. *Mol. Med. Rep.* **1**, 647–650 (2008). 21479463 10.3892/mmr_00000006

[39] Dong, Z. et al. Aberrant expression of Circulating Th17, Th1 and Tc1 cells in patients with active and inactive ulcerative colitis. *Int. J. Mol. Med.* **31**, 989–997 (2013). 23446770 10.3892/ijmm.2013.1287

[40] Bogaert, S. et al. Differential mucosal expression of Th17-related genes between the inflamed colon and ileum of patients with inflammatory bowel disease. *BMC Immunol.* **11**, 61 (2010). 21144017 10.1186/1471-2172-11-61 PMC3016394

[41] Monk, J. M. et al. Th17 cell accumulation is decreased during chronic experimental colitis by (n-3) PUFA in Fat-1 mice. *J. Nutr.* **142**, 117 (2011). 22131549 10.3945/jn.111.147058 PMC3237233

[42] Smallwood, T. B. et al. Helminth Immunomodulation in autoimmune disease. *Front. Immunol.* **8**, 453 (2017). 28484453 10.3389/fimmu.2017.00453 PMC5401880

[43] Anthony, R. M. et al. Memory TH2 cells induce alternatively activated macrophages to mediate protection against nematode parasites. *Nat. Med.* **12**, 955 (2006). 16892038 10.1038/nm1451 PMC1955764

[44] Hartmann, S. & Lucius, R. Modulation of host immune responses by nematode cystatins. *Int. J. Parasitol.* **33**, 1291–1302 (2003). 13678644 10.1016/s0020-7519(03)00163-2

[45] Van der Kleij, D. et al. A novel host-parasite lipid cross-talk. Schistosomal lyso-phosphatidylserine activates toll-like receptor 2 and affects immune polarization. *J. Biol. Chem.* **277**, 48122–48129 (2002). 12359728 10.1074/jbc.M206941200

[46] MAIZELS, R. M., DENHAM, D. A. & BURKE, J. & Phosphorylcholine-bearing antigens in filarial nematode parasites: analysis of somatic extracts, in-vitro secretions and infection Sera from Brugia Malayi and B. pahangi. *Parasite Immunol.* **9**, 49–66 (1987). 2436131 10.1111/j.1365-3024.1987.tb00488.x

[47] Adams, P. N., Aldridge, A., Vukman, K. V., Donnelly, S. & Oneill, S. M. Fasciola hepatica tegumental antigens indirectly induce an M2 macrophage-like phenotype in vivo. *Parasite Immunol.* **36**, 531–539 (2014). 25039932 10.1111/pim.12127

[48] Moradian, A., Abtahi Froushani, S. & Esmaeilnejad, B. Immunomodulatory effects of the somatic antigens of Fasciola Hepatica and Teladorgasia circumcincta in mice immunized with sheep red blood cells. *J. Adv. Biomedical Sci.*10.18502/JABS.V11I3.8784 (2022).

[49] Motomura, Y. et al. Helminth antigen-based strategy to ameliorate inflammation in an experimental model of colitis. *Clin. Exp. Immunol.* **155**, 88–95 (2009). 19016806 10.1111/j.1365-2249.2008.03805.x PMC2665684

[50] Khan, Y. A., Umar, S. & Abidi, S. M. A. Somatic antigens of tropical liver flukes ameliorate Collagen-Induced arthritis in Wistar rats. *PLoS One*. **10**, e0126429 (2015). 25992888 10.1371/journal.pone.0126429 PMC4436316

[51] Tuxun, T. et al. Th17/Treg imbalance in patients with liver cystic echinococcosis. *Parasite Immunol.* **34**, 520–527 (2012). 22803774 10.1111/j.1365-3024.2012.01383.x

[52] Yan, C. et al. The dynamics of Treg/Th17 and the imbalance of Treg/Th17 in Clonorchis sinensis-Infected mice. *PLoS One* **10**, (2015). 10.1371/journal.pone.0143217 PMC4658164 26599407

[53] Vickers, A. J. How many repeated measures in repeated measures designs? Statistical issues for comparative trials. *BMC Med. Res. Methodol.* **3**, 1–9 (2003). 14580266 10.1186/1471-2288-3-22 PMC280679

[54] Lazic, S. E. Four simple ways to increase power without increasing the sample size. *Lab. Anim.* **52**, 621–629 (2018). 29629616 10.1177/0023677218767478

[55] Neurath, M. F. Cytokines in inflammatory bowel disease. *Nat. Rev. Immunol.* **14**, 329–342 (2014). 24751956 10.1038/nri3661

[56] Abraham, C. & Cho, J. H. Inflammatory bowel disease. *N. Engl. J. Med.* **361**, 2066–2078 (2009). 19923578 10.1056/NEJMra0804647 PMC3491806

[57] Tapia-Calle, G. et al. A PBMC-Based system to assess human T cell responses to influenza vaccine candidates in vitro. *Vaccines 2019*. **7**, 181 (2019). 10.3390/vaccines7040181 PMC6963913 31766202

[58] Chen, R. et al. In vitro response of human peripheral blood mononuclear cells (PBMC) to collagen films treated with cold plasma. *Polym. 2017*. **9**, 254 (2017). 10.3390/polym9070254 PMC6431912 30970932

[59] Matsuda, Y., Imamura, R. & Takahara, S. Evaluation of antigen-specific IgM and IgG production during an in vitro peripheral blood mononuclear cell culture assay. *Front Immunol* **8**, (2017). 10.3389/fimmu.2017.00794 PMC5502262 28740496

[60] Wullner, D. et al. Considerations for optimization and validation of an in vitro PBMC derived T cell assay for immunogenicity prediction of biotherapeutics. *Clin. Immunol.* **137**, 5–14 (2010). 20708973 10.1016/j.clim.2010.06.018

[61] Alghanmi, M. et al. Helminth-derived proteins as immune system regulators: a systematic review of their promise in alleviating colitis. *BMC Immunol.* **25**, 1–10 (2024). 38637733 10.1186/s12865-024-00614-2 PMC11025257

